# Evaluation of a Clinical Pathway for Thyroid Nodular Disease: Timings and Delays in the Diagnosis and Treatment of Thyroid Cancer

**DOI:** 10.3390/jcm10235681

**Published:** 2021-12-01

**Authors:** Mildred Sifontes-Dubón, Jose Manuel García-López, Noel González-Ortega, Marcos Pazos-Couselo

**Affiliations:** 1 Doctoral Programme in Medicine Clinical Research, International PhD School of the University of Santiago de Compostela (EDIUS), 15782 Santiago de Compostela, Spain; 2 Endocrinology Department, Mateu Orfila General Hospital, 07703 Mahón, Spain; 3 Endocrinology Department, University Hospital of Santiago de Compostela, 15706 Santiago de Compostela, Spain; Jose.Manuel.Garcia.Lopez@sergas.es; 4 Pathology Department, Mateu Orfila General Hospital, 07703 Mahón, Spain; noelalejandro.gonzalez@hgmo.es; 5 Department of Psychiatry, Radiology, Public Health, Nursing and Medicine, University of Santiago de Compostela, 15782 Santiago de Compostela, Spain; marcos.pazos@usc.es

**Keywords:** delays, clinical pathway, thyroid cancer, thyroid nodule

## Abstract

Background: Due to the high prevalence of nodular thyroid disease in the general population and the need to rule out malignant tumours, a clinical pathway for nodular thyroid disease was created at our tertiary-level hospital. Our study aimed to quantify timings and delays in diagnosis and treatment in this clinical pathway, specifically for patients who were diagnosed with thyroid cancer. Methods: A retrospective review was conducted of patients who were newly diagnosed with thyroid cancer and who had been previously evaluated in the clinical pathway for nodular thyroid disease at our institution during 2015–2017. Patient demographics, previous diagnostic studies, cytological results, tumour details and key dates were analysed to identify wait times in diagnosis and treatment. Results: Forty patients with thyroid cancer were included. The diagnostic delay had a median time of 60 days, and the treatment delay was dependent on cytopathological results. The main cause for delay in the diagnostic phase was the timing of the thyroid ultrasound performed by the radiology department. In the treatment phase, patients with a cytological result of Bethesda III, V or VI underwent surgery at the suggested time, while those in the Bethesda II or IV category did not. Conclusions: The major delay found in the diagnostic phase was the timing of the thyroid ultrasound performed by the radiology department. We are not suggesting that this step must be eliminated, though the implementation of routine ultrasonography in a thyroid clinic can help identify patients who need more urgent evaluation for fine needle aspiration cytology. In our hospital, decision for surgery is based mainly on the cytopathological report. Imaging studies and/or molecular testing could be considered to reduce treatment delays.

## 1. Introduction

Thyroid nodules are common, and the prevalence of these nodules in the general population is high, with the percentage varying depending on the mode of diagnosis from 2–7% by palpation to 19–68% by ultrasonography [1,2,3,4]. The clinical relevance of the evaluation of thyroid nodules is for ruling out thyroid cancer, which occurs in 7–15% of cases depending on age, sex, radiation exposure history, family history and other factors [3]. According to the Surveillance, Epidemiology, and End Results Program, thyroid cancer is responsible for 2.9% of all new cancer cases in the United States of America. Approximately 66.5% of patients with thyroid cancer are diagnosed at the local stage, 28% are diagnosed after the cancer has spread to local lymph nodes and 4% are diagnosed with distant metastasis. The 5-year relative survival rate for localized thyroid cancer is 99.9%, however this percentage falls to 54.9% when the tumour has metastasized [5]. Therefore, undertaking a proper diagnostic workup at an early stage and before treatment is started is crucial to treat this pathology.

Thyroid nodules and multinodular goitres are one of the most common reasons for referral to endocrinology clinics. Their evaluation requires approaches that deal with this highly prevalent disease, approaches which exceed the capacity of conventional care [6]. For this reason, many high-resolution thyroid nodule clinics (HR-TNCs) have been created in several hospitals throughout Spain [7,8,9,10]. The organization of these clinics varies between hospitals according to the needs of every institution and are adapted to the most efficient way to evaluate this pathology [11]. In 2014, a clinical pathway for thyroid nodules that included an HR-TNC was organized in our hospital. This pathway involved endocrinologists, radiologists, pathologists and endocrine surgeons. As described in the literature [12,13], this clinical pathway was implemented to reduce the variation in evaluation, treatment and waiting times, and to focus our attention on those patients with significant findings. By achieving these outcomes, we aimed to reduce cost, improve outcomes and increase satisfaction, as perceived by medical staff and patients. Evaluation of delays could be time-consuming and inefficient due to a high variation in periods of time dependent on the patient activities, the diagnostic procedures and availability of resources and staff, but proactive detection of potentially avoidable delays could help to provide efficient care as a priority for improvement in the healthcare system. The clinical pathway for thyroid nodule assessment in our hospital involves the duration from the first appointment in the endocrinology department under the suspicion or diagnosis of a thyroid nodule to referral to surgery for treatment if necessary.

Our study aimed to quantify timings and delays in diagnosis and treatment in the clinical pathway, focusing on patients who were diagnosed with thyroid cancer.

## 2. Materials and Methods

We performed a retrospective review of patients newly diagnosed with thyroid cancer who had been previously evaluated in the clinical pathway at the University Clinical Hospital of Santiago de Compostela (Santiago, Spain) between 2015 and 2017. All ultrasound-guided fine needle aspiration cytology (UG-FNAC) procedures were performed by the same endocrinologist. All patients underwent surgery by the same endocrine surgery team.

### 2.1. Samples

Fifty patients with thyroid cancer were identified and evaluated during the study period. All included patients who came to the clinical pathway had not been evaluated previously for thyroid pathology in the endocrinology department or were evaluated for nodular pathology or goitre many (more than 5) years earlier. Patients with microcarcinomas were excluded unless the nodule was under study in the HR-TNC, and a puncture was performed. Of the 50 initial patients, 6 were excluded as the nodule reported as thyroid cancer was not the nodule studied in the HR-TNC, they had surgery later on for multinodular goitre or cystic nodules; thyroid cancer was an incidental finding. Another 4 patients were excluded because they were pregnant at the time of first evaluation, and therefore the waiting time until assessment at the HR-TNC was dependent on gestational age. Thus, the study included 40 patients.

The Ethics and Clinical Research Committee of our hospital approved the study protocol, and patient anonymity was preserved.

### 2.2. Description of the Clinical Pathway for Thyroid Nodule Assessment

Patients were referred to the endocrinology department from primary care or other medical or surgical specialties under the diagnosis/suspicion of thyroid nodule or goitre by physical exam or imaging studies (ultrasonography, X-rays, CT scan, MRI and PET). During the first appointment, an endocrinologist performed a proper anamnesis and physical examination. Based on previous radiologic studies, physical exam findings or risks for thyroid cancer, the patients were referred to the HR-TNC. If the patient did not undergo a previous ultrasound, they were either sent to radiology or sent directly to the HR-TNC to have it done. Once the patient came to the HR-TNC, a thyroid ultrasound was performed with an ultrasound system equipped with colour Doppler. The decision to perform FNAC was made according to recommendations of the sonographic patterns, estimated risk of malignancy and fine-needle aspiration guidance for thyroid nodules published by the American Thyroid Association (ATA) in 2015 [3] and/or the British Thyroid Association (BTA) 2014 classification for ultrasound scoring of thyroid nodules [14]. Once the decision was made, UG-FNAC was performed by the same senior endocrinologist. Biopsies were obtained using a 23-gauge needle with a 10 mL syringe under negative pressure. Patients provided signed consent after being fully informed of the procedure. Thyroid cytopathology samples were sent to the pathology department, and the results were reported according to the Bethesda system for reporting thyroid cytopathology [15]. After receiving the cytology report, patients had a follow up visit with the endocrinologist in charge of the patient, and according to cytopathology, radiological report and/or physical exam findings, patients’ diagnoses were commented on by a weekly thyroid committee involving endocrinologists, radiologists, pathologists and endocrine surgeons, to determine the appropriate evaluation and treatment. Patients provided signed consent for surgery before the procedure, when decided upon. 

### 2.3. Data Collection

Data were collected from the registry of patients evaluated in the HR-TNC and electronic records. Study parameters included baseline demographic variables (age, sex), previous radiologic studies, previous FNAC results, the results of UG-FNAC performed in the HR-TNC, anatomopathological reports of thyroidectomy and concordance of the tumour with the studied nodule.

1. To study timing and delay, we collected the following dates:
2. Date of the first visit to the endocrine clinic.
3. Date of thyroid ultrasound performed in the radiology department.
4. Date of referral to the HR-TNC.
5. Date of evaluation in the HR-TNC, when UG-FNAC was performed.
6. Date of the visit to the endocrine clinic with cytological results and referral to surgery.
7. Date of the first visit to the surgery department for evaluation.
8. Date of surgical intervention.

By collecting these dates, we established the following intervals:

- T1: From the date of first evaluation by an endocrine specialist to referral to the HR-TNC for UG-FNAC.
  o T1a: From the date of the first visit to the endocrinology department to the date of thyroid ultrasound performed by the radiology department.
  o T1b: From the date of ultrasound performed by the radiology department to the date of the second evaluation by the endocrinology department for referral to the HR-TNC.
- T2: From the date of referral to the HR-TNC to the date of evaluation for UG-FNAC.
- T3: From the date of UG-FNAC to the date of evaluation at the endocrine clinic with cytological results for referral to surgery.
- T4: From the date of referral to surgery to the date of evaluation by a surgeon.
- T5: From the date of evaluation by a surgeon to the date of thyroid surgery.

By obtaining these intervals, we established:

- Diagnosis delay (DD): defined as the time period between the first endocrinology consultation and the date of evaluation with cytopathological results (from T1 to T3).
- Treatment delay (TD): defined as the time between the visit with cytopathological results and thyroid surgical intervention (from T4 to T5).
- Total waiting time (TWT): defined as the time between the first endocrinology session and surgical intervention (from T1 to T5).

**Figure 1:**
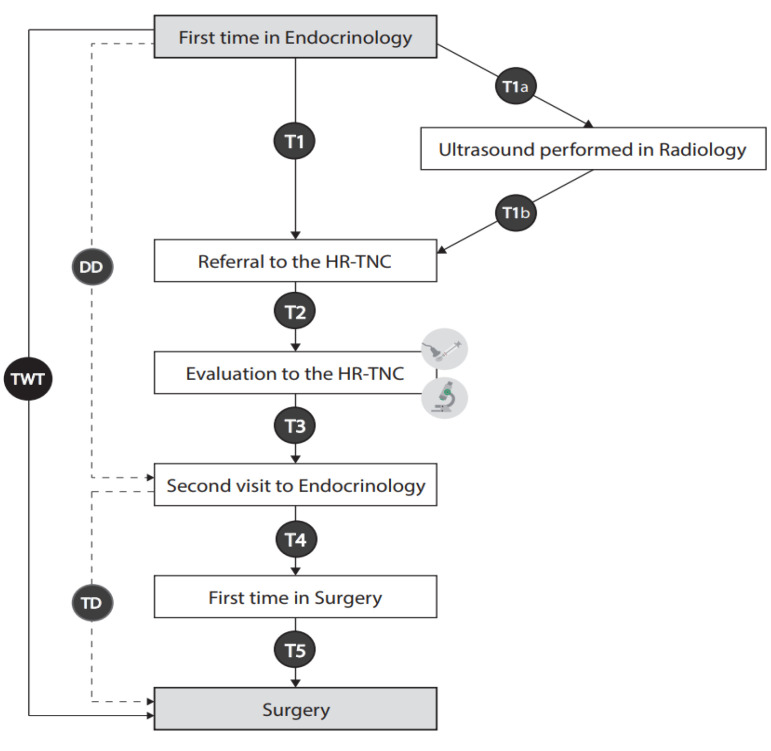
Intervals and clinical pathways for nodular thyroid assessment.

These intervals and the clinical pathway are outlined in Figure 1.

### 2.4. Statistical Analysis

Statistical analysis was performed using SPSS version 22 (SPSS Inc., Chicago, IL, USA). For the estimation of normality, the bias and kurtosis coefficients were used except for those samples where the number of data points was less than 25, in which case the Shapiro–Wilk test was used. Depending on the type of distribution, the arithmetic mean or median was used as a measure of centralization, and the standard deviation or interquartile range was used as a dispersion measure.

## 3. Results

The median timing for diagnosis was approximately 2 months, and the treatment delay was dependent on cytopathological results. Table 1 displays the waiting times for each of the steps in the clinical pathway and the DD, TD and TWT.

The mean age at diagnosis of thyroid cancer in the current study was 48.9 ± 15.7 years, and 75% were female. Thirty-three percent of the patients previously had a thyroid ultrasound before evaluation in the clinical pathway, 37% were sent to the radiology department for a thyroid ultrasound and the rest were referred directly to the HR-TNC for assessment without a previous ultrasound. In the last group, five patients had a different radiologic study (two MRI, three PET/TC) and one had a positive biopsy for thyroid metastasis in the scalp. Seventeen percent of patients had a previous FNAC, most of which were performed many years before coming to the clinic or performed by a private practice (Bethesda I: 3, Bethesda II: 4), and all of these patients underwent a new FNAC in the HR-TNC. The mean size of the studied nodules on ultrasound was 25.2 ± 13 mm.

Sonographic pattern stratification according to the BTA and ATA classification performed at the HR-TNC and their respective cytological results are displayed in Table 2 and Table 3.

Three patients had repeated FNAC: 1 patient had a result of Bethesda I for the first puncture and Bethesda VI for the second puncture, and two patients had a result of Bethesda III for the first puncture and Bethesda IV for the second puncture. 

Correlation between histopathology and cytopathology is outlined in Table 4. 

The mean size of the nodule in the anatomopathological report was 25.2 ± 14.6 mm, concordant with the ultrasound nodule size.

## 4. Discussion

A significant amount of literature has been published on waiting times and delays in the diagnosis and treatment of cancers [16,17,18,19,20,21,22], but there are few data about the timing of diagnosis and treatment of thyroid cancer. To our knowledge, this is one of the few studies in the literature that evaluated this issue.

The main delay found in our data was due to ultrasonography performed by the radiology department, with a median time of 96 days from the time of request to the date of ultrasonography. There is no standardized timing for diagnostic ultrasound, but it usually depends on the capacity of the hospital to perform radiological studies, or the level of suspicion indicated by clinicians upon anamnesis and physical exam. We hypothesized that one possible explanation for this delay might be the high prevalence of thyroid nodules/goitres and the benign nature of most of them. We could not find any other study addressing this issue for comparison. 

Target benchmark for T2 in our hospital was determined by adopting a goal of achieving 30 days from referral to first assessment in the HR-TNC; median time achieved was 26 days. The aim of the HR-TNC at our hospital is to evaluate patients requiring FNAC according to ultrasound characteristics. Thus, it was not feasible to perform a diagnostic ultrasound on all patients coming for thyroid pathology in this clinic; otherwise, the timing for diagnostic cytology would be longer. One possible solution is to include this technique in a routine consultation; this would require proper equipment and training of more endocrinologists in this procedure, as well as longer first appointments. In Spain, many hospitals implement this system [6,7,9,23] and report good results in terms of cost savings and performance of ultrasound and FNAC. Only a study by Castell et al. [9] evaluated delays; with their model, they reported a median time from the first appointment in the endocrinology department to indication for surgery of 35 days. We obtained almost the same result if a patient entered the clinical pathway with a previously performed ultrasound or if a patient was referred directly to the HR-TNC for evaluation. On the other hand, for patients who needed a diagnostic ultrasound once in the clinical pathway, the median time for diagnosis was 166 days. A few other studies compared the timeliness of care of surgeon-performed UG-FNAC vs. radiologist-performed UG-FNAC in patients with thyroid nodules [24,25,26]. These studies only evaluated the period from puncture to diagnosis and reported a shorter delay in the first group as well as a reduction in costs [26]. No studies addressing surgery delays in thyroid pathology were found for comparison. We only found one study involving a survey of otorhinolaryngologists [27] that aimed to assess wait times for thyroid consultations and surgeries based on their opinion and estimations; they reported that an undiagnosed nodule was seen in consultation at 4–8 weeks and a positive/suspicious FNAC at 2–4 weeks. Our results revealed that most patients with nodules categorized as different from benign were seen by surgeons in a median time of less than 4 weeks and that patients with nodules categorized as positive/suspicious were seen in approximately 1 week. The timing of surgery in our data was also found to be dependent on the cytopathological result. The benchmarks for waiting times for surgery in Spain are established according to the royal decree 605/2003 published in the State Official newsletter in 2003 [28] for the national health system, which gives three levels of priority: priority 1: patients whose surgical treatment, being programmable, does not allow a delay longer than 30 days; priority 2: patients whose clinical or social situation permits a relative delay, with an intervention being recommended within less than 90 days; and priority 3: patients whose pathology allows the delay of treatment since it does not produce important sequelae. International references such as target wait times for cancer surgery in Ontario, Canada [29], have designated almost the same benchmarks for surgery as Spain. In our hospital, patients with cytology categorized as Bethesda V and VI are included in the priority 1 category, and patients with cytology characterized as Bethesda II–IV are included in the priority 2 category. Our results revealed that patients with cytology characterized as Bethesda V and VI had surgery in 30 and 32 natural days, respectively. Among those included in the priority 2 category, only those with cytology characterized as Bethesda III had surgery in the recommended time, with a median of 44 days. Patients with cytology characterized as Bethesda IV achieved this time if we counted only working days (median time of 108 natural days), and those with cytology characterized as Bethesda II underwent surgery after a time period that was much longer than the recommendations. It should be noted that the main reason for surgery in the five patients classified as Bethesda II was the nodule size ranging from 30 to 50 mm, three of them with multinodular goitre. For Bethesda III category, one was for the size of the studied nodule and goitre, one had also a 18FDG-PET/TAC uptake and three were solitary nodules and hemithyroidectomy was decided in the thyroid committee. We have better results in delays when FNAC shows a result of Bethesda III, V and VI than in the study by Brake et al. [27], slightly worse when cytology is characterized as Bethesda IV and almost the same delay for benign cytology. However, as mentioned previously, this study was based on a survey, not an assessment of actual delay. BTA guidelines addressed cancer waiting times for patients with suspected cancer, as set out in the Department of Health Cancer Plan document, Cancer waiting targets: a guide. [14] They recommend that the time from “deadline for decision to treat” to “deadline for thyroid surgery” should be 31 days. Although the clinical pathways are not the same, in our study these times could be compared with TD. We obtained a median TD time for Bethesda V–VI of 36 and 41 days, respectively.

Timing for surgery is more consistent when a cytology result is suspicious or diagnostic for thyroid carcinoma; we have found almost the same wait time for surgery as for other head and neck cancers [18]. A challenge remains, however, when a follicular neoplasm or atypia of undetermined significance is reported. One of the reasons for surgery delay in these patients might be related to the estimated risk of malignancy according to the latest update of the Bethesda system [30]: 6–18% and 10–40% for Bethesda III and IV, respectively, if NIFTP is excluded. Another reason could be the low rate of mortality of thyroid cancer compared to other malignancies [31]; in a study by Shin et al. [32], a delay to curative surgery greater than 12 weeks was not associated with increased mortality in patients with thyroid cancer.

Molecular testing could be used to supplement risk assessment in these patients, most recent available versions of commercial molecular tests have demonstrated a positive predicted value varying from 43–68% in Bethesda III, and from 42–82% for Bethesda IV [30,33].

Several limitations need to be considered. It is a small sample and only thyroid cancer patients were included; thus, the general delay for surgery is unknown; however, it may be similar to the findings reported in this study because all patients are treated the same in our national health system. Another limitation is the lack of a cohort for comparison when evaluating delays in the clinical pathway. We hypothesized that timings can be improved; many studies have demonstrated improvements when clinics are well organized. As a relevant strength of our work, it must be stressed that few studies have evaluated delays in a thyroid nodule clinical pathway and surgery timing, and we provided data when addressing these topics. All FNAC procedures were performed by the same endocrinologist; evaluation of cytology and thyroid tissue was performed by the same group of pathologists with a special interest in thyroid pathology; and all thyroidectomies were performed by the same surgery team. This indicates consistency in timing for the most relevant steps in the clinical pathway.

## 5. Conclusions

In conclusion, the main delay found in our study was timing of diagnostic ultrasound; however, we are not suggesting that this step be eliminated. The implementation of routine ultrasonography in a thyroid clinic can help identify patients for whom FNAC is more urgent. The decision to perform surgery in our hospital is based mainly on the cytopathological report. Timing for surgery is accomplished according to recommendations when a nodule is reported as suspicious of malignancy or malignant. Other complementary analyses like imaging studies and/or molecular testing could be considered for patients with Bethesda II, III and IV cytology to reduce the delay to surgery for these groups.

## Figures and Tables

**Table 1:** Wait times from diagnosis to treatment in the clinical pathway.

	*N*	Median (Days)	Q1 (Days)	Q3 (Days)
T1	40	0	0	91
T1a	15	96	22	136
T1b	15	14	12	18
T2	40	26	16	46
T3	39 *	16	8	22
Diagnosis delay	39 *	60	31	166
Ultrasound requested to radiology	15	166	100	265
Previous ultrasound or direct referral to the HR-TNC	24	36	24	65
T4				
Global	39 *	16	7	36
Bethesda I (with lymph node metastasis)	1	76	-	
Bethesda II	4 *	45	33	290
Bethesda III	5	30	19	130
Bethesda IV	14	15	9	34
Bethesda V	6	7	4	10
Bethesda VI	9	7	4	30
T5				
Global	40	39	29	123
Bethesda I (with lymph node metastasis)	1	36	-	-
Bethesda II	5	153	97	186
Bethesda III	5	44	24	148
Bethesda IV	14	108	40	127
Bethesda V	6	30	21	34
Bethesda VI	9	32	19	34
Treatment delay				
Global	39 *	66	38	151
Bethesda I (with lymph node metastasis)	1	112	-	-
Bethesda II	4	181	120	487
Bethesda III	5	176	63	209
Bethesda IV	14	121	62	158
Bethesda V	6	36	32	42
Bethesda VI	9	41	31	61
Total wait time				
Global	40	159	84	289
Bethesda I (with lymph node metastasis)	1	212		
Bethesda II	5	224	183	593
Bethesda III	5	238	95	554
Bethesda IV	14	243	128	317
Bethesda V	6	84	69	116
Bethesda VI	9	94	65	143

* Data missing for 1 patient.

**Table 2:** BTA nodule sonographic patterns and correlation with cytopathological results according to the Bethesda system.

	U5 Malignant	U4 Suspicious	U3 Indeterminate Equivocal	U2 Benign	Not Classified	Total (%)
Bethesda VI	2	1	4	2	0	9 (22.5%)
Bethesda V	1	0	2	3	0	6 (15%)
Bethesda IV	0	3	2	8	1	14 (35%)
Bethesda III	0	0	2	3	0	5 (12.5%)
Bethesda II	1	0	1	3	0	5 (12.5%)
Bethesda I	0	1	0	0	0	1 (2.5%)
Total (%)	4 (10%)	5 (12.5%)	11 (27.5%)	19 (47.5%)	1 (2.5%)	40 (100%)

**Table 3:** ATA nodule sonographic patterns and correlation with cytopathological results according to the Bethesda system.

	High Suspicion	Intermediate Suspicion	Low Suspicion	Very Low Suspicion	Not Classified *	Total
Bethesda VI	2	4	0	1	2	9 (22.5%)
Bethesda V	1	1	1	1	2	6 (15%)
Bethesda IV	0	2	5	0	7	14 (35%)
Bethesda III	0	1	2	0	2	5 (12.5%)
Bethesda II	1	0	1	1	2	5 (12.5%)
Bethesda I	0	0	0	0	1	1 (2.5%)
Total	4 (10%)	8 (20%)	9 (22.5%)	3 (7.5%)	16 (40%)	40 (100%)

* ATA scoring was used from 2016.

**Table 4:** Histopathology correlation with cytopathology results according to the Bethesda system.

	Papillary Thyroid Carcinoma	Follicular Thyroid Carcinoma	Oncocytic (Hurthle Cell) Carcinoma	NIFPT	Anaplastic Carcinoma	Total
Bethesda VI	9	0	0	0	0	9 (22.5%)
Bethesda V	6	0	0	0	0	6 (15%)
Bethesda IV	9	3	1	1	0	14 (35%)
Bethesda III	4	1	0	0	0	5 (12.5%)
Bethesda II	2	0	1	1	1	5 (12.5%)
Bethesda I	1	0	0	0	0	1 (2.5%)
Total	31 (77.5%)	4 (10%)	2 (5%)	2 (5%)	1 (2.5%)	40 (100%)

## Data Availability

The datasets used and/or analysed during the current study are available from the corresponding author on reasonable request.

## Abbreviations

HR-TNC	High-resolution thyroid nodule clinic.
UG-FNAC	Ultrasound-guided fine needle aspiration cytology.
FNAC	Fine-needle aspiration cytology.
DD	Diagnostic delay.
TD	Treatment delay.
TWT	Total waiting time.
